# Differences in Receipt of Three Preventive Health Care Services by Race/Ethnicity in Medicare Advantage Plans: Tracking the Impact of Pay for Performance, 2010 and 2013

**DOI:** 10.5888/pcd13.160047

**Published:** 2016-09-08

**Authors:** Daniel H. Jung, Mari Palta, Maureen Smith, Thomas R. Oliver, Eva H. DuGoff

**Affiliations:** Author Affiliations: Mari Palta, Maureen Smith, Thomas R. Oliver, Eva H. DuGoff, University of Wisconsin School of Medicine and Public Health, Madison, Wisconsin.

## Abstract

**Introduction:**

In 2012, the Centers for Medicare and Medicaid Services (CMS) introduced the Quality Bonus Payment Demonstration, a pay-for-performance (P4P) program, into Medicare Advantage plans. Previous studies documented racial/ethnic disparities in receipt of care among participants in these plans. The objective of this study was to determine whether P4P incentives have affected these disparities in Medicare Advantage plans.

**Methods:**

We studied 411 Medicare Advantage health plans that participated in the Medicare Health Outcome Survey in 2010 and 2013. Preventive health care was defined as self-reported receipt of health care provider communication or treatment to reduce risk of falling, improve bladder control, and monitor physical activity among individuals reporting these problems. Logistic regression stratified by health care plan was used to examine racial/ethnic disparities in receipt of preventive health care before and after the introduction of the P4P program in 2012.

**Results:**

We found similar racial/ethnic differences in receipt of preventive health care before and after the introduction of P4P. Blacks and Asians were less likely than whites to receive advice to improve bladder control and more likely to receive advice to reduce risk of falling and improve physical activity. Hispanics were more likely to report receiving advice about all 3 health issues than whites. After the introduction of P4P, the gap decreased between Hispanics and whites for improving bladder control and monitoring physical activity and increased between blacks and whites for monitoring physical activity.

**Conclusion:**

Racial/ethnic differences in receipt of preventive health care are not always in the expected direction. CMS should consider developing a separate measure of equity in preventive health care services to encourage health plans to reduce gaps among racial/ethnic groups in receiving preventive care services.

**MEDSCAPE CME**

Medscape, LLC is pleased to provide online continuing medical education (CME) for this journal article, allowing clinicians the opportunity to earn CME credit.

This activity has been planned and implemented through the joint providership of Medscape, LLC and *Preventing Chronic Disease*. Medscape, LLC is accredited by the Accreditation Council for Continuing Medical Education (ACCME), the Accreditation Council for Pharmacy Education (ACPE), and the American Nurses Credentialing Center (ANCC), to provide continuing education for the healthcare team.

Medscape, LLC designates this Journal-based CME activity for a maximum of 1.00 ***AMA PRA Category 1 Credit(s)™***. Physicians should claim only the credit commensurate with the extent of their participation in the activity.

All other clinicians completing this activity will be issued a certificate of participation. To participate in this journal CME activity: (1) review the learning objectives and author disclosures; (2) study the education content; (3) take the post-test with a 75% minimum passing score and complete the evaluation at http://www.medscape.org/journal/pcd; (4) view/print certificate.

**Release date: September 8, 2016; Expiration date: September 8, 2017**

**Learning Objectives**

Upon completion of this activity, participants will be able to:

1. Evaluate the overall impact of P4P incentives on receipt of preventive care before and after implementation of P4P incentives in 2012, and overall findings regarding racial/ethnic disparities
2. Distinguish disparities in receipt of preventive care between Asians and blacks vs whites
3. Distinguish disparities in receipt of preventive care between Hispanics vs whites

**EDITOR**

Rosemarie Perrin

Editor, *Preventing Chronic Disease*

Disclosure: Rosemarie Perrin has disclosed no relevant financial relationships.

**CME AUTHOR**

Laurie Barclay, MD

Freelance writer and reviewer, Medscape, LLC

Disclosure: Laurie Barclay, MD, has disclosed the following relevant financial relationships:

Owns stock, stock options, or bonds from: Pfizer

**AUTHORS**

Daniel H. Jung, BS

University of Wisconsin School of Medicine and Public Health, Madison, Wisconsin

Disclosure: Daniel H. Jung, BS, has disclosed no relevant financial relationships.

 

Mari Palta, PhD

University of Wisconsin School of Medicine and Public Health, Madison, Wisconsin

Disclosure: Mari Palta, PhD, has disclosed no relevant financial relationships.

 

Maureen Smith, MD, PhD

University of Wisconsin School of Medicine and Public Health, Madison, Wisconsin

Disclosure: Maureen Smith, MD, PhD, has disclosed no relevant financial relationships.

 

Thomas R. Oliver, PhD

University of Wisconsin School of Medicine and Public Health, Madison, Wisconsin

Disclosure: Thomas R. Oliver, PhD, has disclosed no relevant financial relationships.

 

Eva H. DuGoff, PhD

University of Wisconsin School of Medicine and Public Health, Madison, Wisconsin

Disclosure: Eva H. DuGoff, PhD, has disclosed no relevant financial relationships.

 

## Introduction

Preventive health care can reduce illness or injury, detect chronic disease, and control the effects of disease. The Affordable Care Act increased access to preventive care in private health insurance plans and in the Medicare program by eliminating patient cost sharing for some services (1). Few studies have examined the delivery of preventive care in Medicare Advantage plans, which provide health care coverage to 31% of Medicare beneficiaries through private health plans (2).

Medicare Advantage plan performance has been publicly reported since 2008 and has been subject to pay-for-performance (P4P) assessment under the Quality Bonus Payment Demonstration since 2012 (3). The Centers for Medicare and Medicaid Services (CMS) collect performance data from Medicare Advantage plans using 3 data sources — the Healthcare Effectiveness Data and Information Set, Consumer Assessment of Healthcare Providers and Systems, and the Medicare Health Outcomes Survey — with 48 performance measures to assess plan performance (2). Using data from 2006, Ng and colleagues found significant differences in the delivery of preventive care services between black and white patients (4). They reported that black patients were more likely to receive care from their health care provider to reduce falls, but white patients were more likely to receive osteoporosis care. Race was not associated with receiving urinary incontinence care or physical activity advice.

The objective of our study was twofold: 1) to provide information on the status of racial/ethnic differences in the receipt of clinically recommended preventive care in 3 areas: reducing risk of falling, improving bladder control, and monitoring physical activity, and 2) to assess whether the introduction of P4P incentives in 2012 affected rates of receipt of this care. Our a priori hypotheses were that 1) racial/ethnic minority seniors (adults aged ≥65 y) were less likely to receive these 3 preventive health care services and 2) the introduction of P4P would not affect disparities in the receipt of these services. 

## Methods

We compared data on Medicare Advantage plan participants enrolled in 411 plans who responded to the Medicare Health Outcomes Survey in 2010 and 2013. We conducted a retrospective study that used time-series data to model the difference-in-difference, by race/ethnicity, after the introduction of P4P in the Medicare Advantage program in the receipt of 3 preventive health care services: 1) reducing risk of falling, 2) improving bladder control, and 3) monitoring physical activity. These 3 performance measures are also endorsed by the National Quality Forum and were developed by the National Committee for Quality Assurance as dependent variables to assess receipt of appropriate preventive health care among survey respondents aged 65 years or older (8).

### Data

The Medicare Health Outcomes Survey, a survey of Medicare beneficiaries enrolled in private health plans, is used by CMS to monitor health plan performance (5,6). It uses an overlapping panel design that surveys at 2-year intervals a random sample of health plan members drawn from Medicare Advantage plan contracts with at least 500 enrollees (7). We defined eligible respondents as Medicare beneficiaries who completed 80% or more of the survey and who reported their race/ethnicity. A total of 546,931 people were eligible for the survey in 2010, of whom 54.5% responded; 597,134 were eligible for the survey in 2013, of whom 44.4% responded. We focused on respondents enrolled in one of the 411 plans of 602 possible plans offered in both 2010 (471 plans) and 2013 (502 plans). We excluded enrollees in a plan that was offered only in 2010 (42,786 people excluded) or 2013 (37,553 people excluded). We also excluded people who self-identified as Native Hawaiian or other Pacific Islander or as American Indian or Alaska Native (8,123 in 2010 and 8,060 in 2013) because of small sample sizes for each performance measure. The analytic sample for each performance measure for both years consisted of respondents eligible for each measure based on age or health history: reducing risk of falling (n = 149,773), improving bladder control (n = 113,650), and monitoring physical activity (n = 383,207).

### Variables

For respondents who reported a problem with falling, walking, or balancing, appropriate care was defined as a yes response to the survey question “Has your doctor or other health provider done anything to help prevent falls or treat problems with balance or walking?” Appropriate care for improving bladder control was assessed by a yes or no response from respondents who reported a urine leakage problem to the survey question “There are many ways to treat urinary incontinence, including bladder training, exercises, medication, and surgery. Have you received these or any other treatments for your current urine leakage problem?” Appropriate monitoring of physical activity was defined as a yes response to the survey question “In the past 12 months, did a doctor or other health provider advise you to start, increase, or maintain your level of exercise or physical activity?”

Our primary predictors of interest were race/ethnicity and the time variable to distinguish before and after the introduction of P4P. We used self-reported race/ethnicity instead of CMS race, which was classified as non-Hispanic white (hereafter, white), non-Hispanic black (hereafter, black), Asian, and Hispanic. Because the P4P program was finalized in 2011 and implemented in 2012, we used 2010 as the baseline year and 2013 to assess racial/ethnic differences in care (9).

Additional covariates were selected according to the Aday–Andersen health behavior model, which provides a widely accepted framework that conceptualizes the effect of the relationship between individual factors, medical care factors, and environmental factors on a person’s health care use and health outcomes (10). Available predisposing factors were age (65–69 y, 70–74 y, 75–79 y, 80–84 y, ≥85 y), sex, and geographic region of residence (Box). Available enabling factors were annual income (<$20,000, ≥$20,000, or not reported), and education (<high school diploma, high school or general equivalency diploma, >high school diploma, or not reported). Health was assessed by self-report (poor/fair/good, very good/excellent, or not reported), by body mass index (BMI) (kg/m^2^) (<20, 20–25, 25–30, >30, or not reported), by self-reported difficulty with activities of daily living (ADLs) (ie, bathing, dressing, eating, getting in or out of chairs, walking, or using the toilet; none, ≥1, or not reported) or inability to perform them, and number of chronic conditions based on 13 self-reported conditions (0–1, ≥2, or not reported).

**Box. States Included in Regions, Centers for Medicare and Medicaid Services**

**Table:** 

Region	States
Region 1	Connecticut, Maine, Massachusetts, New Hampshire, Rhode Island, Vermont
Region 2	Puerto Rico, Virgin Islands, New York, New Jersey
Region 3	Maryland, District of Columbia, Delaware, West Virginia, Virginia, Pennsylvania
Region 4	North Carolina, South Carolina, Tennessee, Florida, Georgia, Alabama, Kentucky, Mississippi
Region 5	Michigan, Minnesota, Ohio, Illinois, Indiana, Wisconsin
Region 6	Texas, Louisiana, Arkansas, Oklahoma, New Mexico
Region 7	Missouri, Kansas, Iowa, Nebraska
Region 8	North Dakota, Utah, South Dakota, Wyoming, Colorado, Montana
Region 9	Nevada, American Samoa, Arizona, California, Guam, Hawaii, Northern Mariana Islands
Region 10	Washington, Alaska, Idaho, Oregon

### Statistical analysis

After describing the characteristics of respondents eligible for each performance measure, we calculated differences in receipt of the 3 services between 2010 and 2013 by race/ethnicity. We examined the receipt of each service separately by using a multivariable logistic model stratified by health plan and fit via conditional logistic regression:

Logit (π_ij_) = α_i_ + β_1_Race_ij_ + β_2_Time_ij_ + β_3_Race_ij_ × Time_ij_ + β_4_Z_ij_

where *i* designates health care plan and *j* designates individual within the plan.

We were interested in the observed racial/ethnic disparities for receipt of each service and the change in the disparities over time, controlling for a vector of individual-level covariates Z_ij_. Therefore, we calculated the odds ratios for race/ethnicity in 2010 (exp [β_1_]) and in 2013 (exp[β_1_ + β_2_]). The interaction term between race/ethnicity and time (exp[β_3_]) is the difference in receipt of each service between racial/ethnic minorities and non-Hispanic whites in 2013 compared with 2010. Conditional logistic models that stratified by health plan automatically adjusted for all potential confounders for plan-level characteristics and limited the analysis to plans present in both years. We used the *proc logistic* function in SAS 9.4 (SAS Institute Inc) to conduct the conditional logistic regression analysis. We adjusted for age, sex, region of residence, income, educational level, number of ADLs and chronic conditions, BMI, and self-reported health.

We performed 2 sensitivity tests to assess the robustness of our results. We ran logistic regression models on individuals who were enrollees in any Medicare Advantage plan in either 2010 or 2013. We also ran multilevel logistic regression models that accounted for clustering by health plan using random effects.

## Results

Across all 3 measures, more than 75% of eligible respondents reported having multiple chronic conditions (Table 1). Nearly one-third reported having one or more limitations in an ADL for monitoring physical activity, and more than half reported having one or more limitations in ADL for reducing risk of falling and improving bladder control. Of all respondents, across performance measures, 65.5% to 68.7% were enrolled in a health maintenance organization, 26.3% to 29.3% were in a preferred provider organization, and nearly 75% were enrolled in a for-profit health plan.

**Table 1:** Participant Characteristics, Study of Differences in Receipt of Preventive Health Care Services by Race/Ethnicity for Three Care Measurements in Medicare Advantage Plans, Medicare Health Outcomes Survey, 2010 and 2013^a^

Characteristic	Reducing Risk of Falling (N = 149,773)		Improving Bladder Control (N = 113,650)		Monitoring Physical Activity (N = 383,207)	
	2010 (N = 79,202)	2013 (N = 70,571)	2010 (N = 60,346)	2013 (N = 53,304)	2010 (N = 204,642)	2013 (N = 178,565)
**Age, y**						
65–69	19,173 (24.2)	17,351 (24.6)	14,778 (24.5)	12,795 (24.0)	61,537 (30.1)	52,759 (29.5)
70–74	17,223 (21.7)	16,198 (23.0)	13,549 (22.5)	12,663 (23.8)	52,373 (25.6)	47,838 (26.8)
75–79	15,938 (20.1)	13,758 (19.5)	12,339 (20.4)	10,613 (19.9)	40,650 (19.9)	34,215 (19.2)
80–84	13,302 (16.8)	11,357 (16.1)	9,886 (16.4)	8,524 (16.0)	28,019 (13.7)	24,075 (13.5)
≥85	13,566 (17.1)	11,907 (16.9)	9,79 (16.2)	8,709 (16.3)	22,063 (10.8)	19,678 (11.0)
**Sex**						
Female	50,557 (63.8)	45,021 (63.8)	43,027 (71.3)	38,022 (71.3)	119,440 (58.4)	105,302 (59.0)
**Race/ethnicity** b						
White	58,736 (74.2)	44,564 (63.1)	47,497 (78.7)	35,633 (66.8)	155,299 (75.9)	115,815 (64.9)
Black	7,179 (9.1)	5,181 (7.3)	4,631 (7.7)	3,336 (6.3)	17,422 (8.5)	12,328 (6.9)
Asian	2,411 (3.0)	2,364 (3.3)	1,687 (2.8)	1,645 (3.1)	7,570 (3.7)	7,177 (4.0)
Hispanic	10,876 (13.7)	18,462 (26.2)	6,531 (10.8)	12,690 (23.8)	24,351 (11.9)	43,245 (24.2)
**Medicare insurance plan**						
Health maintenance organization	53,538 (67.6)	48,513 (68.7)	39,821 (66.0)	35,905 (67.4)	134,013 (65.5)	119,647 (67.0)
Preferred provider organization	21,582 (27.2)	18,550 (26.3)	17,195 (28.5)	14,598 (27.4)	60,024 (29.3)	49,961 (28.0)
Other	4,082 (5.2)	3,508 (5.0)	3,330 (5.5)	2,801 (5.3)	10,605 (5.2)	8,957 (5.0)
**Plan’s tax status**						
For profit	57,952 (73.2)	50,918 (72.2)	43,909 (72.8)	38,238 (71.7)	150,656 (73.6)	130,746 (73.2)
**Education**						
Did not graduate from high school	24,329 (30.7)	20,101 (28.5)	16,335 (27.1)	13,472 (25.3)	53,285 (26.0)	43,028 (24.1)
High school or general equivalency diploma	26,597 (33.6)	21,995 (31.2)	21,465 (35.6)	17,585 (33.0)	71,525 (35.0)	57,510 (32.2)
More than high school	27,325 (34.5)	26,748 (37.9)	21,871 (36.2)		77,579 (37.9)	73,809 (41.3)
No response	951 (1.2)	1,727 (2.4)	675 (1.1)	1,302 (2.4)	2,253 (1.1)	4,218 (2.4)
**Annual income, $**						
<20,000	32,787 (41.4)	27,754 (39.3)	23,573 (39.1)	19,636 (36.8)	69,084 (33.8)	56,794 (31.8)
≥20,000	32,635 (41.2)	28,970 (41.1)	26,432 (43.8)	23,405 (43.9)	97,249 (47.5)	85,249 (47.7)
Not reported	13,780 (17.4)	13,847 (19.6)	10,341 (17.1)	10,263 (19.3)	38,309 (18.7)	36,522 (20.5)
**Number of chronic conditions**						
0 or 1	8,145 (10.3)	7,689 (10.9)	7,766 (12.9)	7,184 (13.5)	46,609 (22.8)	42,883 (24.0)
≥2	71,057 (89.7)	62,880 (89.1)	52,580 (87.1)	46,117 (86.5)	158,028 (77.2)	135,679 (76.0)
Not reported	0 (0.0)	2 (0.0)	0 (0.0)	3 (0.0)	5 (0.0)	3 (0.0)
**Physical limitations in activities of daily living**						
0	25,605 (32.3)	22,450 (31.8)	25,886 (42.9)	22,693 (42.6)	127,163 (62.1)	111,064 (62.2)
≥1	53,534 (67.6)	48,063 (68.1)	34,408 (57.0)	30,570 (57.4)	77,309 (37.8)	67,360 (37.7)
No response	63 (0.1)	58 (0.1)	52 (0.1)	41 (0.1)	170 (0.1)	141 (0.1)
**Self-reported general health**						
Excellent or very good	11,988 (15.1)	11,233 (15.9)	11,974 (19.8)	11,134 (20.9)	63,177 (30.9)	58,140 (32.6)
Good or poor	65,887 (83.2)	57,989 (82.2)	47,426 (78.6)	41,194 (77.3)	138,453 (67.7)	117,274 (65.7)
Not reported	1,327 (1.7)	1,349 (1.9)	946 (1.6)	976 (1.8)	3,012 (1.5)	3,151 (1.8)
**Body mass index (kg/m^2^)**						
≤20 (Underweight)	5,156 (6.5)	3,736 (5.3)	3,376 (5.6)	2,420 (4.5)	11,491 (5.6)	8,413 (4.7)
20–25 (Normal)	20,405 (25.8)	16,563 (23.5)	15,135 (25.1)	12,070 (22.6)	57,618 (28.2)	46,617 (26.1)
26–30 (Overweight)	26,640 (33.6)	23,203 (32.9)	20,451 (33.9)	17,376 (32.6)	76,610 (37.4)	64,578 (36.2)
≥30 (Obese, morbidly obesity)	25,870 (32.7)	24,156 (34.2)	20,548 (34.1)	19,181 (36.0)	56,096 (27.4)	51,467 (28.8)
Not reported	1,131 (1.4)	2,913 (4.1)	836 (1.4)	2,257 (4.2)	2,827 (1.4)	7,490 (4.2)

a All values are number (percentage).

b Self-reported race/ethnicity was used instead of Center for Medicare and Medicaid Services race.

Overall, the adherence rate for receipt of care ranged in 2010 from 32.3% for improving bladder control to 67.2% for reducing risk of falling and ranged in 2013 from 31.3% for improving bladder control to 71.2% for reducing risk of falling in 2013 (Table 2). Between 2010 and 2013, receipt of care to reduce risk of falling increased slightly across all racial/ethnic groups. Receipt of care to improve bladder control decreased, and receipt of care to monitor physical activity increased for whites, blacks, and Asians.

**Table 2:** Likelihood by Race/Ethnicity^a^ of Receiving Three Preventive Care Services Among Enrollees in Medicare Advantage Plans Who Participated in Medicare Health Outcomes Survey, 2010 and 2013

Characteristic	Adherence Rate, %		Conditional Logistic Regression^b^, OR (95% CI) [*P* c]		
	2010	2013	2010^d^	2013^d^	Ratio^e^
**Reducing risk of falling**					
White	55.8	57.9	1 [Reference]	1 [Reference]	1 [Reference]
Black	67.2	71.2	1.34 (1.26–1.42) [<.001]	1.40 (1.30–1.50) [<.001]	1.04 (0.96–1.14) [.32]
Asian	67.4	67.8	1.34 (1.21–1.49) [<.001]	1.23 (1.11–1.37) [<.001]	0.92 (0.80–1.05) [.21]
Hispanic	64.1	65.5	1.10 (1.05–1.17) [<.001]	1.05 (1.01–1.10) [.01]	0.95 (0.90–1.01) [.13]
**Improving bladder control**					
White	36.0	35.9	1 [Reference]	1 [Reference]	1 [Reference]
Black	32.3	31.3	0.89 (0.83–0.96) [<.001]	0.85 (0.79–0.92) [<.001]	0.95 (0.86–1.06) [.36]
Asian	35.7	32.3	0.99 (0.88–1.10) [.08]	0.87 (0.77–0.98) [.02]	0.88 (0.76–1.02) [.10]
Hispanic	38.3	34.4	1.16 (1.08–1.23) [<.001]	1.01 (0.97–1.06) [.59]	0.88 (0.82–0.94) [<.001]
**Monitoring physical activity**					
White	46.3	48.5	1 [Reference]	1 [Reference]	1 [Reference]
Black	50.7	54.5	1.19 (1.14–1.23) [<.001]	1.26 (1.21–1.31) [<.001]	1.06 (1.01–1.12) [.02]
Asian	52.9	55.5	1.42 (1.35–1.50) [<.001]	1.49 (1.41–1.57) [<.001]	1.05 (0.98–1.13) [.17]
Hispanic	53.8	50.8	1.33 (1.28–1.37) [<.001]	1.14 (1.11–1.17) [<.001]	0.86 (0.83–0.89) [<.001]

Abbreviations: CI, confidence interval; OR, odds ratio.

a Self-reported race/ethnicity was used instead of Centers for Medicare and Medicaid Services race.

b Regressions included controls for age, sex, region (see Box), income, education level, body mass index, self-reported health, number of chronic conditions, and number of limitations on activities of daily living.

c *P* values calculated using a conditional logistic regression model.

d Odds ratio for each performance measure for racial/ethnic minorities versus non-Hispanic whites.

e Ratio of odds ratio for racial/ethnic minorities versus non-Hispanic white between 2013 and 2010.

In both 2010 and 2013, blacks were more likely than whites to report receiving care to reduce the risk of falling and to monitor physical activity but were less likely to report receiving care for improving bladder control (Table 2). Asians were also more likely than whites to report receiving care to reduce risk of falling and monitor physical activity. However, Asians were less likely than whites to report receiving care for improving bladder control in 2013. Hispanics were more likely than whites to report receiving care for all 3 measures in 2010 and for 2 out of 3 measures (reducing the risk of falling and monitoring physical activity) in 2013.

We found that the gap in care between Hispanics and whites decreased between 2010 and 2013 for services to improve bladder control and monitoring physical activity and did not change for reducing the risk of falling. The gap between blacks and whites increased for monitoring physical activity and did not change significantly for reducing risk of falling or improving bladder control. We found no significant changes in the gap between Asians and whites.

In sensitivity tests, we found some differences from the main analysis. Data from all reporting health plans showed that the gap in care to improve bladder control decreased between Asians and whites (OR, 0.85; 95% CI, 0.74–0.98; *P* = .03), and the gap for monitoring physical activity increased between Hispanics and whites (OR, 1.06; 95% CI, 1.01–1.11; *P* < .01) between 2010 and 2013. Analyses using multilevel logistic regression models showed that the gap in care to reduce risk of falling decreased between Hispanics and whites (OR, 0.90; 95% CI, 0.84–0.97; *P* < .01), and the gap for monitoring physical activity between blacks and whites was not significant (OR, 1.04; 95% CI, 0.99–1.10; *P* = .15).

## Discussion

Rates of preventive care services to reduce the risk of falling, improve bladder control, and monitor physical activity were persistently low among Medicare Advantage program participants who responded to the Medicare Health Outcomes Survey. These preventive care services could forestall adverse events and prevent reduced quality of life. Falls are estimated to affect one in 3 seniors, leading to serious injuries, hospital admission, or death (8). Urinary incontinence is a persistently underdiagnosed condition estimated to affect as many as half of seniors (11,12). Studies have found that urinary incontinence is associated with negative physical and mental health outcomes (11). Physical activity is associated with lowered risk of developing chronic conditions such as cardiovascular disease, diabetes, and depression (13). However, we found that fewer than half of Medicare Advantage plan enrollees who were eligible for these services and who participated in the Medicare Health Outcomes Survey were not receiving them.

We found some differences in care among racial/ethnic minorities. Racial/ethnic minorities were more likely to report receiving care for reducing risk of falling and monitoring physical activity. The observed gaps for reducing risk of falling and monitoring physical activity were not in the expected direction, which was that whites would be more likely to receive care. Studies by CMS and RAND Corp also found several health services where gaps in care by race/ethnicity were different than expected, that is, that whites would be more likely to receive care for monitoring physical activity and reducing risk of falling (14,15).We found no evidence that the introduction of P4P in 2012 significantly improved receipt of preventive care or reduced racial/ethnic disparities in care related to improving bladder control. Racial/ethnic minority seniors were more likely to report receiving care to reduce risk of falling and monitor physical activity than whites before and after the introduction of P4P. Hispanic seniors were more likely to report receiving all 3 services than white seniors before the introduction of P4P and more likely to receive 2 services (reducing the risk of falling and monitoring physical activity) after the introduction of P4P.

Previous studies assessing the effect of P4P on racial/ethnic disparities among Medicare beneficiaries focused on inpatient services and reported no significant effect (16,17). We extended this research to participants in Medicare Advantage programs and found that P4P incentives did not improve racial/ethnic disparities in care for urinary incontinence. Reports on disparities among Medicare Advantage participants by income and disability status (18) and by race/ethnicity (4) indicated that socioeconomically disadvantaged populations were not always less likely to receive health care services than higher-income populations. Our findings confirm previous reports that differences in quality of care by race/ethnicity were not always in the expected direction, that is, poorer for racial/ethnic minorities than for whites. This study showed that Asians and Hispanics were also more likely than whites to receive clinically recommended care for reducing risk of falling and monitoring physical activity.

Although we found some racial/ethnic disparities in preventive care among participants in Medicare Advantage programs, both in 2010 and 2013, P4P did not produce noticeable improvement in the rates of receiving preventive care services between racial/ethnic groups. After the introduction of P4P, the gap increased between black and white seniors in care to monitor physical activity and was unchanged between Asians and whites. Although the gap between Hispanics and whites decreased on measures for urinary incontinence and physical activity, bivariate results indicated that this was due to a decrease in receipt of care by Hispanics. These results highlight the importance of tracking the impact of financial incentives such as P4P on care delivery to monitor both intended and unintended consequences.

Our studies had limitations. First, we used self-reported data to measure quality of care, which are subject to recall bias. Second, our measure of the burden of chronic disease quantified the number of conditions but did not account for levels of disease severity. However, we also measured physical limitations that would identify people with physical disabilities. Third, our data did not include number of physician visits, which would be likely to increase the probability of receiving clinically recommended services. Last, we were not able to assess concordance in patient–provider language, which may have played a role in receipt of preventive care services.

Despite these limitations, this study presented novel evidence on the Quality Bonus Payment Demonstration and its impact on racial/ethnic disparities in the receipt of clinically recommended care. We found little evidence that the introduction of P4P reduced the gaps in quality of care. Furthermore, we found little evidence that P4P improved the delivery of preventive care services. One possible explanation for the absence of P4P’s effect on health care disparities may be the lack of performance measures designed to assess racial/ethnic disparities. The use of more than 40 different performance measures may also have diluted the importance of preventive care measures.

The results of this study suggest that whether P4P rewards alone will improve health disparities in care is uncertain. CMS should consider modifying the P4P structure to focus health plan attention on addressing known disparities in care. CMS could do this by increasing the weight of performance measures designed to assess racial/ethnic disparities. CMS recently started publicly reporting health plan performance by racial/ethnic groups but not for the 3 measures we examined in this study (15). Reporting on health care by racial/ethnic group is a step to create incentives for Medicare Advantage plans to address gaps in care. As a next step, CMS should consider developing a separate measure of health care equity that could hold health plans accountable for reducing gaps in preventive care services among racial/ethnic groups. We hope our research will inform policymakers, health plans, and health care providers about the state of racial/ethnic disparities in the receipt of preventive health care in the Medicare Advantage program performance measures and the effect of P4P on these disparities.

## Post-Test Information

To obtain credit, you should first read the journal article. After reading the article, you should be able to answer the following, related, multiple-choice questions. To complete the questions (with a minimum 75% passing score) and earn continuing medical education (CME) credit, please go to http://www.medscape.org/journal/pcd. Credit cannot be obtained for tests completed on paper, although you may use the worksheet below to keep a record of your answers. You must be a registered user on Medscape.org. If you are not registered on Medscape.org, please click on the "Register" link on the right hand side of the website to register. Only one answer is correct for each question. Once you successfully answer all post-test questions you will be able to view and/or print your certificate. For questions regarding the content of this activity, contact the accredited provider, CME@medscape.net. For technical assistance, contact CME@webmd.net. American Medical Association’s Physician’s Recognition Award (AMA PRA) credits are accepted in the US as evidence of participation in CME activities. For further information on this award, please refer to http://www.ama-assn.org/ama/pub/about-ama/awards/ama-physicians-recognition-award.page. The AMA has determined that physicians not licensed in the US who participate in this CME activity are eligible for ***AMA PRA Category 1 Credits™***. Through agreements that the AMA has made with agencies in some countries, AMA PRA credit may be acceptable as evidence of participation in CME activities. If you are not licensed in the US, please complete the questions online, print the AMA PRA CME credit certificate and present it to your national medical association for review.

## Post-Test Questions

### Study Title: Differences in Receipt of Three Preventive Health Care Services by Race/Ethnicity in Medicare Advantage Plans: Tracking the Impact of Pay for Performance, 2010 and 2013

#### CME Questions

1. You are advising a large health maintenance organization regarding correction of racial/ethnic disparities in receipt of preventive care. According to the Centers for Medicare and Medicaid Services (CMS) survey study by Jung and colleagues, which of the following statements about the overall impact of pay-for-performance (P4P) incentives on receipt of preventive care before and after implementation of P4P incentives in 2012 and overall findings regarding racial/ethnic disparities is ***correct***?
  - Adherence rate for receipt of preventive care improved significantly from 2010 to 2013
  - Whites were more likely to receive care than racial/ethnic minorities for reducing risk of falling and monitoring physical activity
  - CMS should consider a separate measure of equity in preventive healthcare services to encourage health plans to reduce gaps among racial/ethnic groups
  - Between 2010 and 2013, receipt of care to reduce risk of falling decreased across all racial/ethnic groups
2. According to the CMS survey study by Jung and colleagues, which of the following statements about disparities in receipt of preventive care between Asians and blacks vs whites is ***correct***?
  - Blacks and Asians were more likely than whites to receive advice to improve bladder control
  - Blacks and Asians were less likely than whites to receive advice to reduce risk of falling and improve physical activity
  - After the introduction of P4P, the gap decreased between blacks and whites for monitoring physical activity
  - After the introduction of P4P, there were no significant changes in the gap between Asians and whites
3. According to the CMS survey study by Jung and colleagues, which of the following statements about disparities in receipt of preventive care between Hispanics vs whites is ***correct***?
  - Between 2010 and 2013, the gap in care between Hispanics and whites decreased for services to improve bladder control and monitor physical activity
  - Between 2010 and 2013, the gap in care between Hispanics and whites decreased for reducing the risk of falling
  - Hispanics were less likely than whites to report receiving advice about reducing the risk of falling
  - Changes in the gap between Hispanics and whites for bladder control and falling risk were due to increased receipt of care by Hispanics

**Table:** Evaluation

**1. The activity supported the learning objectives.**				
**Strongly Disagree**				**Strongly Agree**
1	2	3	4	5
**2. The material was organized clearly for learning to occur.**				
**Strongly Disagree**				**Strongly Agree**
1	2	3	4	5
**3. The content learned from this activity will impact my practice.**				
**Strongly Disagree**	** **	** **	** **	**Strongly Agree**
1	2	3	4	5
**4. The activity was presented objectively and free of commercial bias.**				
**Strongly Disagree**				**Strongly Agree**
1	2	3	4	5
